# Surgical site infections in Italian Hospitals: a prospective multicenter study

**DOI:** 10.1186/1471-2334-8-34

**Published:** 2008-03-07

**Authors:** Nicola Petrosillo, Cecilia MJ Drapeau, Emanuele Nicastri, Lorena Martini, Giuseppe Ippolito, Maria Luisa Moro, ANIPIO

**Affiliations:** 1 2^nd ^Infectious Diseases Division, National Institute for Infectious Diseases "L. Spallanzani", Via Portuense, 292-00149 Rome, Italy; 2 4^th ^Infectious Diseases Division, National Institute for Infectious Diseases "L. Spallanzani", Via Portuense, 292-00149 Rome, Italy; 3 Epidemiology Department, National Institute for Infectious Diseases "L. Spallanzani", Via Portuense, 292-00149 Rome, Italy; 4 Agenzia Sanitaria Regione Emilia-Romagna, Viale Aldo Moro, 21-40127 Bologna, Italy; 5 ANIPIO: Italian Association of Infection Control Professionals: Full names and institutional addresses of the ANIPIO members are cited in the acknowledgment section

## Abstract

**Background:**

Surgical site infections (SSI) remain a major clinical problem in terms of morbidity, mortality, and hospital costs. Nearly 60% of SSI diagnosis occur in the postdischarge period. However, literature provides little information on risk factors associated to in-hospital and postdischarge SSI occurrence. A national prospective multicenter study was conducted with the aim of assessing the incidence of both in-hospital and postdisharge SSI, and the associated risk factors.

**Methods:**

In 2002, a one-month, prospective national multicenter surveillance study was conducted in General and Gynecological units of 48 Italian hospitals. Case ascertainment of SSI was carried out using standardized surveillance methodology. To assess potential risk factors for SSI we used a conditional logistic regression model. We also reported the odds ratios of in-hospital and postdischarge SSI.

**Results:**

SSI occurred in 241 (5.2%) of 4,665 patients, of which 148 (61.4%) during in-hospital, and 93 (38.6%) during postdischarge period. Of 93 postdischarge SSI, sixty-two (66.7%) and 31 (33.3%) were detected through telephone interview and questionnaire survey, respectively. Higher SSI incidence rates were observed in colon surgery (18.9%), gastric surgery (13.6%), and appendectomy (8.6%). If considering risk factors for SSI, at multivariate analysis we found that emergency interventions, NNIS risk score, pre-operative hospital stay, and use of drains were significantly associated with SSI occurrence. Moreover, risk factors for total SSI were also associated to in-hospital SSI. Additionally, only NNIS, pre-operative hospital stay, use of drains, and antibiotic prophylaxis were associated with postdischarge SSI.

**Conclusion:**

Our study provided information on risk factors for SSI in a large population in general surgery setting in Italy. Standardized postdischarge surveillance detected 38.6% of all SSI. We also compared risk factors for in-hospital and postdischarge SSI, thus providing additional information to that of the current available literature. Finally, a large amount of postdischarge SSI were detected through telephone interview. The evaluation of the cost-effectiveness of the telephone interview as a postdischarge surveillance method could be an issue for further research.

## Background

Surgical site infections (SSI) remain a major clinical problem in terms of morbidity, mortality [1], length of stay and hospital costs [2-4]. Nearly 60% of SSI diagnosis, ranging from 21 to 100%, [5-9] occur after hospital discharge and the trend increases as the length of postoperative hospital stay is getting shorter and the number of one day surgery procedures enlarges over time.

According to current literature, active SSI surveillance is useful in reducing SSI incidence by surveillance-induced infection control efforts [10-12]. However, although representing a methodological tool of increasing importance for its large impact on SSI rates [5,13-16], it also represents a methodological challenge for clinicians and epidemiologists, because the patient is not constantly under medical observation, and microbiological investigation becomes difficult to perform after discharge.

Indeed, a matter of concern is the choice of the most effective postdischarge surveillance (PDS) methodology to estimate SSI rates; actually, the incidence of postdischarge SSI could depend on which of the different PDS method is being performed. The authors of a recent systematic review of PDS methods reported that no valid and reliable method of SSI case ascertainment is available till now [17]. Most of the studies on postdischarge SSI aimed also to evaluate the associated risk factors, and interestingly, they suggested that most of the predictors of in-hospital SSI were not predictors of postdischarge SSI [7]. Particularly, the composite National Nosocomial Infection Surveillance (NNIS) risk score was found to be positively correlated to the risk of inhospital SSI [18], whereas other reports have documented a discrepancy between the predicted risk of infection by NNIS index score and the incidence of postdischarge SSI [7,8].

A national prospective multicenter study was conducted with the aim of assessing the incidence of both in-hospital and postdisharge SSI, and the associated risk factors. We aimed also to evaluate the performance of the NNIS risk index in predicting SSI occurrence in the Italian Surgical setting, and its validity in stratifying risk according to specific surgical procedures.

## Methods

In 2002, a one-month, prospective national multicenter surveillance study was conducted in General and Gynecological units of 48 Italian hospitals on 4,665 consecutive patients undergoing the following surgical procedures: hernia repair (n = 1,079; 23.1%), caesarean section (n = 1,050; 22.5%), cholecystectomy (n = 781;16.7%), breast surgery (n = 436; 9.3%), colon surgery (n = 364; 7.8%), gastric surgery (n = 165; 3.5%), abdominal hysterectomy (n = 355; 7.6%), vaginal hysterectomy (n = 171;3.6%), appendectomy (n = 238; 5.1%), vascular surgery (n = 16; 0.3%), and other minor interventions on genitourinary tract (n = 10; 0.21%). The following 6 surgical procedures included also the laparoscopic technique: cholecistectomy (n = 438; 56%), appendectomy (n = 24; 10%), colon surgery (n = 18; 4.9%), abdominal hysterectomy (n = 15; 4.2%), hernia repair (n = 39; 3.6%), and gastric surgery (n = 6; 3.6%). Table 1 summarizes the main characteristics of the study population. All the above listed procedures were included in the surveillance program.

**Table 1:** Population and operation specific characteristics

**Total number of patients** **Males (%)**	4,665 1630 (34.9%)		
	
	Mean	Median	Range
**Age, years**	53	54	(18; 99)
**ASA score**	2	2	(1;5)
**Length of preoperative stay, days**	2	1	(0; 69)
**Length of postoperative stay, days**	6	4	(0; 377)
**Duration of intervention, hours**	1.3	1	(0.2; 8.5)
**NNIS index score**	0	0	(-1*; 3)

-1 refers to interventions without risk factors for the NNIS index score, performed laparoscopically.

ASA = American Society of Anesthesiologists.

NNIS = National Nosocomial Infection Surveillance.

One month before starting the study, for each participating center, referent infection control personnel, including infection control practitioners, physicians, and nursing staff attended a training meeting on how to collect information and on patients' follow-up standardization.

### In-hospital SSI

For each patient, infection control personnel used a questionnaire to collect information on demographic and operation specific characteristics, and on SSI occurrence. The following information was ascertained: demographic characteristics, dates of admission and discharge, operation characteristics (i.e. type, date, and duration of the surgical procedure, emergency or elective surgical procedures, wound contamination class according to the four-point wound infection score of the National Research Council [19], American Society of Anesthesiologists (ASA) physical status score [20], any use of endoscopic surgical approach, any prosthesis implant), antimicrobial perioperative prophylaxis, presence of drains, occurrence, date of onset and type of in-hospital SSI.

SSI diagnosis was performed using the Centers for Diseases Control and Prevention (CDC) NNIS standardized criteria [21]. According to these criteria, SSI are classified as being either incisional or organ/space. Incisional SSI are divided into those involving skin and subcutaneous tissue (superficial incisional) and those involving deeper soft tissue of surgical incision (deep incisional SSI). Organ/space SSI involve any part of the anatomy other than incised body wall layers, that was opened or manipulated during an operation [21].

Moreover, the NNIS risk index score [22] was calculated by assigning one point each for a contaminated wound according to CDC definition [19], an ASA score ≥ 3, and surgical procedures lasting longer than the NNIS-derived 75th percentile for the duration of the procedure; whenever the procedure was done laparoscopically, the NNIS score was modified by subtracting 1 point, as more recently suggested by Gaynes et al. [23]. The laparoscopic surgical procedures without risk factors for the NNIS index score, where included in a new risk category defined as "M".

### Postdischarge SSI

The last section of the questionnaire was dedicated to the PDS, which reported information collected within 30 days after the operative procedure. Case ascertainment of postdischarge SSI was carried out using the following active surveillance methods: 1) a follow-up questionnaire, that was given to the patient to be filled during follow-up visits by the hospital infection control personnel; if the patient was visited in another health care facility the physician was asked to fill out the questionnaire and to send it back to the reference center; 2) patients who missed follow-up visits were called by telephone by the infection control nurses to be interviewed for any wound signs and symptoms suggestive of SSI. In the first case, SSI were identified by using the CDC criteria [21]. For patients surveilled by telephone calling, SSI diagnosis was based on criteria that are included in CDC definition of SSI, at least for superficial infections. These criteria included one or more of the following self-reported conditions: 1) purulent wound secretion; 2) pain or tenderness, localized swelling, redness, or heat at the surgical site.

To assess potential risk factors for SSI we performed a univariate analysis which included the following variables: gender, age, emergency interventions, NNIS score, any prosthesis placement, preoperative hospital stay, use of drains, any perioperative antimicrobial prophylaxis. All the covariates with a p-value less than 0.1 at univariate analysis were included in a multivariate conditional logistic regression model.

### Statistical analysis

Multivariate statistical analysis was performed using SPSS (Version 11.0 Chigago Ilinois USA) statistical software; the continuous variables were analyzed by calculating the variance while the dichotomous variables were analyzed by using the Χ^2 ^test. Results were expressed in terms of Odds Ratio (OR) with their respective 95% Confidence Interval (CI).

Each participating institution (see in the acknowledgment section) sought ethical clearance through their own Ethical Committee according to local regulations. Verbal informed consent was obtained.

## Results

Surgical site infections occurred in 241 (5.2%) of 4,665 patients. One-hundred-forty-eight SSI (61.4%) occurred during hospital stay, and 93 (38.6%) within 30 days after discharge. Of the 148 in-hospital SSI, 87, 37, and 24 were classified as superficial, deep, and organ/space, respectively. Post-discharge SSI observed at medical follow-up were all superficial. Those SSI ascertained by telephone calling were not classified according to the CDC classification, due to lack of direct medical observation. However, the telephone interviewers referred that in about 90% of cases SSI could be classified as superficial.

Table 2 reports the frequencies of SSI according to surgical procedure and NNIS risk index category. If excluding the procedures with unknown PDS status, the highest SSI incidence rates were observed in colon surgery (18.9%), gastric surgery (13.6%), and appendectomy (8.6%); the remaining surgical procedures had lower and similar SSI rates (range 3.2–4%).

**Table 2:** Frequencies of SSI^§ ^according to type of intervention and NNIS^§§ ^index category.

Surgical procedure*	Total** number of procedures (n = 4,386)	**N SSI (%)**	**N SSI (%) according to NNIS index category**			
			**M**	**0**	**1**	**2 + 3**
**Breast surgery**	406	13 **(3.2%)**	-	10/281 **(3.5%)**	2/111 **(1.8%)**	1/10 **(10%)**
**Cesarean section**	1012	27 **(2.6%)**	-	20/775 **(2.6%)**	6/218 **(2.7%)**	0/8 **(0%)**
**Cholecistectomy**	740	30 **(4%)**	4/287 **(1.4%)**	11/236 **(4.6%)**	9/145 **(6.2%)**	4/59 **(6.7%)**
**Gastric surgery**	147	20 **(13.6%)**	1/3 **(33.3%)**	3/50 **(6%)**	1/63 **(1.6%)**	4/23 **(17.4%)**
**Appendectomy**	221	19 **(8.6%)**	0/16 **(0%)**	1/108 **(0.9%)**	13/76 **(17.1%)**	3/18 **(16.6%)**
**Colon surgery**	338	64 **(18.9%)**	2/4 **(50%)**	14/70 **(20%)**	24/151 **(15.9%)**	22/109 **(20.2%)**
**Hernia repair**	1014	41 **(4%)**	1/19 **(5.3%)**	20/641 **(3.1%)**	15/283 **(5.3%)**	4/46 **(8.7%)**
**Abdominal hysterectomy**	346	21 **(6.1%)**	1/12 **(8.3%)**	7/217 **(3.2%)**	11/91 **(12.1%)**	1/20 **(5%)**
**Vaginal hysterectomy**	162	6 **(3.7%)**	-	3/117 **(2.5%)**	3/41 **(7.3%)**	0/2 **(0%)**

^§ ^Surgical site infections.

^§§^National Nosocomial Infection Surveillance index category.

*Cardiovascular and other minor interventions are not reported because the denominator was too little (n = 21) to be included in statistical analysis.

** The procedures with no postdischarge surveillance (258) were not included.

M: no risk factor for NNIS index score plus laparoscopical procedure (NNIS = -1).

When calculating the OR of SSI for each NNIS risk index category, significant differences were observed between NNIS index category 0 and 1 for appendectomy: OR 24.38, 95% CI 3.21–510.30; p < 0.001, and for abdominal hysterectomy: OR 3.74, 95% CI 1.34–10.62; p = 0.003, and between NNIS index category M and >M for cholecistectomy: NNIS score 0: OR 3.46; 95% CI 1.00–13.06; p = 0.026; NNIS score 1: OR 4.68; 95% CI 1.29–18.41; p = 0.005; NNIS score 2 + 3: OR 5.15; 95% CI 1.04–25.39; p = 0.012.

There was no significant difference between SSI rates in laparoscopic and open cholecistectomy (15/438; 3.4% vs. 15/339; 4.4%; OR 0.77; 95% CI 0.35–1.68; p = 0.473). For the other surgical procedures which also used the laparoscopic technique (i.e. gastric surgery, appendectomy, colon surgery, hernia repair, and abdominal hysterectomy), differences among the SSI rates between NNIS index category M and >M were not statistically significant.

We found that 4,486 out of 4,665 enrolled patients, excluding those who developed an in-hospital SSI (n = 148), and those with a postoperative in-hospital stay longer than 30 days (n = 31), were eligible for PDS. A total of 4,228/4,486 (94.2%) patients underwent PDS, whereas 258 (5.8%) patients were lost to follow-up. One hundred and eighty-four questionnaires were filled and returned back to the reference center; telephone interviews were carried out for the remaining 4,041 patients. Sixty-two (66.7%) and 31 (33.3%) of 93 postdischarge SSI were detected through telephone interview and questionnaire survey, respectively.

At univariate analysis, age ≥ 55 years, emergency interventions, NNIS score > 0, use of prosthesis, pre-operative hospital stay > 1 day, and use of drains resulted significantly associated with SSI occurrence. At multivariate analysis, emergency interventions, NNIS score > 0, pre-operative hospital stay > 1 day, and use of drains remained significantly associated with SSI occurrence (Table 3).

**Table 3:** Risk factors for surgical site infection (SSI).

			**Univariate analysis**		**Multivariate analysis**	
**Variable**	**Total §**	**No. SSI/total§**	**Odds Ratio (95% CI^^)**	p*	**Odds Ratio (95% CI^^)**	p*

**Gender**						
Male	4,406	97/1521	1.30 (0.99–1.70)	0.054	1.13 (0.83–1.55)	0.429
Female		144/2,885				
**Age, years**						
18–36	4,172	35/1,101	1			
37–54		45/1,004	1.43 (0.89–2.30)	0.118	1.11 (0.72–1.69)	0.635
55–70		67/1,107	1.96 (1.27–3.04)	0.001	0.91 (0.63–1.31)	0.622
>70		88/960	3.07 (2.02–4.69)	<0.001	1.51 (0.91–2.51)	0.113
**Emergency Interventions**						
Yes	4,396	69/944	1.50 (1.11–2.03)	0.005	1.73 (1.22–2.44)	0.002
No		172/3,452				
**NNIS score**						
M	4,330	8/341	1			
0		90/2,507	1.55 (0.72–3.48)	0.237	1.18 (0.77–1.82)	0.441
1		90/1,182	3.43 (1.59–7.71)	<0.001	1.82 (1.14–2.90)	0.012
2 + 3		41/300	6.59 (2.91–15.50)	<0.001	3.34 (1.41–7.93)	0.006
**Prosthesis**						
Yes	4,393	23/762	0.60 (0.39–0.91)	0.011	0.62 (0.38–1.04)	0.07
No		218/3,631				
**Pre-operative hospital stay, days**						
	4,337	130/3,136	2.15 (1.63–2.83)	<0.001	1.45 (1.06–1.98)	0.02
0–1		102/1,201				
>1						
**Drains**						
No	4,406	97/2,894	1			
1–3 days		31/702	3.04 (2.31–3.99)	<0.001	2.39 (0.65–1.65)	<0.001
>3 days		111/810	4.58 (3.41–6.15)	<0.001	2.17 (1.38–3.43)	<0.001
**Perioperative prophylaxis**						
Yes	4,406	213/3,857	0.92 (0.60–1.40)	0.684	-	-
No		28/549				

§ total number of procedures considered not equal to the study cohort (n = 4,665) for missing data; also patients with missing data for SSI occurrence during postdischarge (n = 258) were excluded from the analysis.

^^ 95% confidence intervals (CI).

* p-value.

M no risk factor for NNIS index score + laparoscopical procedure (NNIS = -1).

NNIS = National Nosocomial Infection Surveillance.

When stratifying risk factors for in-hospital and postdischarge SSI, we found that those factors that were significantly associated for total SSI, were also associated for in-hospital SSI. However, if considering postdischarge SSI, only NNIS >1, pre-operative hospital stay >1, use of drains, and antibiotic prophylaxis were associated with postdischarge SSI (Table 4).

**Table 4:** Risk factors for in-hospital and postdischarge surgical site infection (SSI).

	**In-hospital period**			**Post-discharge period**		
**Variable**	**Total §**	**No. SSI/total§**	**Odds Ratio (95% CI^^)**	**Total§S**	**No. SSI/total §§**	**Odds Ratio (95% CI^^)**

**Gender**						
Male	4,665	67/1,625	1.57 (1.12–2.21)*	4,258	30/1,454	0.92 (0.58–1.45)#
Female		81/3,040			63/2,804	
**Age (years)**						
18–36		15/1,163	1		20/1,086	1
37–54	4,420	26/1,057	1.93 (0.98–3.84)#	4,027	19/978	1.06 (0.54–2.08)#
55–70		44/1,162	3.01 (1.62–5.69)*		23/1,063	1.18 (0.62–2.25)#
>70		60/1,038	4.70 (2.58–8.68)*		28/900	1.71 (0.93–3.18)#
**Emergency Intervention**						
	4,655			4,258		
Yes		51/1,002	1.97 (1.37–2.82)*		18/903	0.89 (0.51–1.53)#
No		97/3,653			75/3,355	
**NNIS score §**						
		4/358	1		4/336	1
M		38/2,621	1.30 (0.44–4.32)#		52/2,459	1.79 (0.62–5.87)#
	4,579			4,166		
0		65/1,260	4.81 (1.67–		25/1,108	1.92 (0.63–6.54)#
1		30/340	15.63)*		11/263	3.62 (1.05–
2 + 3			11.42 (3.86–38)*			13.65)*
**Prosthesis**						
Yes	4,652	6/801	0.20 (0.08–0.46)*	4,245	17/756	1.03 (0.58–1.80)#
No		142/3,851			76/3,489	
**Pre-operative hospital stay (days)**	4,589			4,196		
		72/3,300			58/3,064	
0–1		69/1,289	2.54 (1.79–3.60)*		33/1,132	1.56 (0.99–2.45)*
>1						
**Drains**						
No		41/3,045	1		56/2,853	1
1–3 days	4,665	19/764	1.87 (1.04–3.33)*	4,261	12/686	0.89 (0.45–1.72)#
>3 days		88/856	8.40 (5.66–12.48)*		23/722	1.64 (0.97–2.75)*
**Perioperativ e antibiotic prophylaxis**	4,665			4,258		
Yes		139/4,071	0.44 (0.21–0.89)*		74/3,718	1.80 (1.04–3.07)*
No		9/594			19/540	

§ total number of procedures surveilled during hospital stay not equal to the study cohort (n = 4,665) for missing data.

NNIS = National Nosocomial Infection Surveillance.

§§ total number of interventions surveilled during post-discharge, does not include missing data for SSI occurrence during postdischarge (n = 258), and patients with in-hospital SSI (n = 148) or with postoperative hospital stay ≥ 30 days (n = 25).

^^ 95% confidence intervals.

* p-value < 0.05.

# p value not significant.

M: no risk factor + endoscopical procedure.

Moreover, clean or clean/contaminated operations such as breast surgery, caesarean section, cholecistectomy, and hernia repair had postdischarge SSI rates from 1.2 to 3.3 times higher than those observed during the hospital stay. On the other hand, we found that contaminated operations had higher in-hospital SSI rates (Table 5).

**Table 5:** Rates of in-hospital and postdischarge surgical site infections (SSIs) according to type of intervention.

**Surgical procedure**	N°Postdischarge SSI/total* (n = 4,407)	**In-hospital SSI/total (n = 4,665)**	**Posdischarge/In-hospital ratio**
**Vascular surgery**	0/16 **(0%)**	0/16 **(0%)**	-
**Breast surgery**	10/406 **(2.4%)**	3/437 **(0.7%)**	**3.4**
**Cesarean section**	19/1012 **(1.8%)**	8/1050 **(0.7%)**	**2.5**
**Cholecistectomy**	16/740 **(2.1%)**	14/781 **(1.8%)**	**1.2**
**Gastric surgery**	5/147 **(3.4%)**	15/165 **(9.1%)**	0.4
**Appendectomy**	3/221 **(1.4%)**	16/238 **(6.7%)**	0.2
**Colon surgery**	6/338 **(1.8%)**	58/364 **(15.9%)**	0.1
**Hernia repair**	23/1014 **(2.3%)**	18/1079 **(1.6%)**	**1.4**
**Abdominal hysterectomy**	8/346 **(2.3%**)	13/355 **(3.7%)**	**0.6**
**Vaginal hysterectomy**	3/162 **(1.8%)**	3/171 **(1.7%)**	1
**Other genitourinary surgery**	1/5 **(20%)**	0/5 **(0%)**	-

* The procedures with unknown PDS status (n = 258) were not included.

## Discussion

In our study we used the NNIS methodology in order to standardize and compare our data with those published in the current literature. Indeed, according to a retrospective review on European studies [4], the true rate of SSI is actually unknown in Europe and is likely to have been underestimated as a consequence of the variability of data collection, surveillance methods, and type of surgical procedures that are investigated.

In the present study, the overall SSI incidence rate was 5.2% which is lower than that reported in other Italian (range 5.4%–12.8%) [24-26,9], and European [7,15] studies including the European Surveillance of Surgical site Infections HELICS – Improving patient Safety in Europe (IPSE) network [27], which used the NNIS definitions and surveillance methodology. In particular, if considering only general, gynecological (including caesarean section), and vascular surgery interventions, European SSI rates are higher and globally range from 6.34% to 14.8% [7,15].

However, for some types of interventions our SSI rates are higher than those reported in the literature. In our study, we found that the highest SSI incidence were observed in colon surgery (18.9%), gastric surgery (13.6%) and appendectomy (8.6%). These rates are about two fold higher than those reported by other national and European studies, and by the U.S. NNIS reports [10,9,24,28]. Moreover, the rates of SSI after cholecystectomies was very high, as compared to those mentioned in the HELICS network [27] These differences could depend on type, accuracy, and distribution of PDS methods [10,15,24,10], on losses in follow-up, and on the impossibility to assess the postdischarge response validity.

In our study, we found that emergency interventions, NNIS index score, and pre-operative hospital stay were independently associated to overall SSI occurrence. These findings are not discordant with previous reports of the literature [18,22,24]; however, we provided additional details that deserve particular attention.

First, when stratifying by NNIS index category according to operative procedure, we found no significant differences between NNIS index category, for most of the procedures except for appendectomy, abdominal hysterectomy (Table 2). Regarding laparoscopic procedures, it should be noticed that the small number of these procedures in some interventions was a limiting factor in the statistical analysis.

Actually, conflicting data are reported in the literature about the predictive power of the NNIS index score, at least for specific interventions such as cesarean sections and breast interventions [10,8,29-31]. Furthermore, in agreement with our findings, in other reports [32,22] NNIS index score did not perform well for single surgical procedures, but it was found to perform better when taking into account a group of different interventions. On the contrary, according to the HELICS-IPSE most recent reports, the SSI increases with the NNIS index score, even for caesarean sections [27]. A possible explanation of our finding may be that the NNIS index score underestimates SSI incidence as it represents an inpatient risk score which does not include postdischarge surveillance variables. Therefore, NNIS index score may not represent a suitable tool for the SSI risk evaluation in particularly for clean interventions where SSI are likely to be detected during postdischarge surveillance, probably due to anticipated hospital discharge.

Second, we observed that the use of drains was significantly associated with SSI occurrence independently of the length of drainage (i.e., 1–3 or < 3 days); additionally, we found that length of preoperative hospital stay > 1 day was associated with a significant risk of SSI. Of note, pre-operative hospital may be independent from one day surgery procedures. This finding underlines the importance of performing day surgery procedures, whenever possible.

Third, we found the SSI rate among laparoscopic cholecistectomy interventions was lower than the open technique but did not reached a statistical significance, contrarily to what reported in other reports [33].

In the present study, we also aimed to provide information on postdischarge SSI. Interestingly, we found that 38.6% of SSI were diagnosed after discharge; this rate is comparable to the 34.8% reported by Fiorio et al. [24] in Italian general surgery inpatients, although other similar surveillance studies [7-9] found considerably higher postdischarge SSI rates, ranging from 34.8 to 60%. We also observed that the higher ratio of postdischarge/in-hospital SSI was found in clean interventions, as evidenced in other recent reports [7,9,15]. In particular, breast interventions, and cesarean sections had higher SSI incidence rates during the postdischarge period, ie 2.4% and 1.8%, respectively (Table 5). A possible explanation to this finding could be the shorter postoperative stay which characterizes the above mentioned interventions. Our finding should advice infection control practitioners of the need for improving PDS surveillance methods for targeted surgical procedures.

Moreover, we were able to provide a better insight on the risk factors for postdischarge SSI, for which information from the literature is scant. Indeed, in our study we found that most predictors of in-hospital SSI were not predictors of postdischarge SSI, as already suggested by Delgado-Rodriguez et al. [7] Only NNIS >1, preoperative hospital stay, and antibiotic prophylaxis were significantly associated with postdischarge diagnosis. The presence of drains was marginally predictive of postdischarge SSI, and only if drains remained for more than 3 days. These data are discordant with those of other similar studies [7,5] that failed to identify risk factors for postdischarge SSI, particularly on what concerns the validity of the NNIS index score.

Another important finding of our study is the importance of telephone interview for the detection of postdischarge SSI. Indeed, almost 60% of postdischarge SSI were diagnosed by telephone interview, which underlines the importance of this cost-effective surveillance methodology for SSI detection. On the other hand, we are aware of the little supporting evidence regarding the validity and reliability of self-reported diagnosis, as suggested by other reports [17].

Our study has some limitations including the lack of other possible host-related risk factors to be included in multivariate analysis such as body mass index, malnutrition, diabetes, cancer, immunosuppressive drugs. Moreover, another limitation of our study depends on the lack of standardized post-discharge surveillance methodologies, which could affect the validity of the post-discharge SSI rate. In fact, accurate, and standardized methods for defining and monitoring post-discharge SSI are needed to correct assess infection rates.

Additionally, our study did not provide any information regarding the cost-effectiveness of telephone post-discharge surveillance, and on the economic impact of postdischarge SSI on public health. We suggest to evaluate the cost-effectiveness of such postdischarge method for further research.

Finally, although most of the postdischarge SSI were superficial, we think that looking at them could make sense in terms of economic impact on public health as evidenced by recent studies [34,35].

## Conclusion

Our study provided information on risk factors for SSI occurrence in a large population in general surgery setting in Italy. Moreover, standardized postdischarge surveillance methodology was carried out, which detected 38.6% of all SSI. We were also able to compare risk factors for in-hospital and postdischarge SSI, thus providing additional information to that of the current available literature. Particularly, we found that NNIS >1, preoperative hospital stay >1 day, use of drains, and antibiotic prophylaxis were significantly associated to postdischarge SSI diagnosis. Moreover, a large amount of postdischarge SSI were detected through telephone interview, which underlines the importance of this PDS methodology for its cost-effectiveness.

## Competing interests

Financial competing interests: NP is on the speakers' bureau for several companies including Merck Sharpe & Dohme, Aventis, Ethicon, GSK, Pfizer, AstraZeneca, Roche, Gilead. None of the other authors has financial competing interests. None of the authors has non financial competing interests to disclose.

## Authors' contributions

NP, LM, and MLM conceived the study, contributed to its design, and to the manuscript draft and final version. CMJD contributed to the analysis of the data and to manuscript draft and final version. EN performed statistical analysis, and contributed to interpretation of data. ANIPIO group contributed to data collection. All authors read and approved the final manuscript.

## Pre-publication history

The pre-publication history for this paper can be accessed here:


